# Endoplasmic reticulum stress response pathway-mediated cell death in ovarian cancer

**DOI:** 10.3389/fonc.2024.1446552

**Published:** 2024-09-10

**Authors:** Qiaochu Chen, Chan Li, Wei Wei, Jia Li, Fangyuan Liu, Yuqian Fu, Liping Tang, Fengjuan Han

**Affiliations:** ^1^ Department of Obstetrics and Gynecology, Heilongjiang University of Chinese Medicine, Harbin, China; ^2^ Department of Obstetrics and Gynecology, The First Affiliated Hospital of Heilongjiang University of Chinese Medicine, Harbin, China; ^3^ The Affiliated Tumor Hospital of Harbin Medical University, Harbin, China

**Keywords:** ovarian cancer, endoplasmic reticulum stress response pathway, organelle, cell biology, cell death, potential therapeutic strategies

## Abstract

The endoplasmic reticulum (ER) is one of the largest organelles, and Endoplasmic Reticulum Stress Response Pathway is a series of responses triggered by the homeostatic imbalance of the ER and the state in which unfolded or misfolded proteins accumulate in the ER, which can trigger cell death. Cell death plays a crucial role in the development of diseases such as gynecological oncology. Herein, we review the current research on the response and ovarian cancer, discussing the key sensors (IRE1, PERK, ATF6), and the conditions under which it occurs (Ca^2+^ homeostasis disruption, hypoxia, others). Using the response as a starting point, provide a comprehensive overview of the relationship with the four types of cell death (apoptosis, autophagy, immunogenic cell death, paraptosis) in an attempt to provide new targeted therapeutic strategies for the organelle-Endoplasmic Reticulum Stress Response Pathway-cell death in ovarian cancer therapy.

## 1 Introduction

Ovarian Cancer (OC) is one of the malignant cancers of the reproductive system, with an incidence of 3.4% and a mortality rate of 4.7%, making it the sixth most common malignant cancer worldwide (1, 2). Moreover, due to its low early diagnosis rate, high recurrence rate, susceptibility to chemotherapy resistance, and other characteristics, the clinical treatment of this cancer faces a difficult challenge. Therefore, further understanding of the mechanisms that lead to the development of OC and identifying new therapeutic targets may help improve the prognosis of OC patients.

Cells are the most basic units of life activities, and the membrane-bound structural organelles with specific morphology and functions are the functional regions of eukaryotic cells that execute life activities. Therefore, exploring the research situation related to OC cells based on organelles is especially crucial. The Endoplasmic Reticulum (ER) is a classic membranous organelle in which the processing, modification, and folding of proteins are essential regulatory processes that dominate cell function, fate, and death and can participate in the growth and metastasis of cancer-like diseases mediated by changes in the Endoplasmic Reticulum Stress Response Pathway (3). Endoplasmic Reticulum Stress Response Pathway refers to the fact that under the influence of hypoxia, Ca^2+^ homeostasis, and other conditions, proteins in ER are misfolded and continuously accumulated, finally causing Endoplasmic Reticulum Stress (ERS) and thus activating the Unfolded Protein Response (UPR) (4). Activation of UPR is closely related to survival regulation, angiogenesis, invasion, metastasis, and drug resistance of cancer cells (5). In addition, recent studies have also identified the Endoplasmic Reticulum Stress Response Pathway as a key regulator of the ovarian follicular microenvironment, which plays a pivotal role in various pathological states of the ovary. As a result, this response pathway is emerging as an attractive therapeutic target for cancer (6).

When cells are subjected to ERS, activated UPR leads to two opposite outcomes (7). On the one hand, cells experiencing short-term ERS exhibit enhanced ER capacity, which restores ER homeostasis to a physiological state through UPR-processed proteins, allowing the cells to survive. On the other hand, cells experiencing long-term ERS exhibit severely impaired physical capacity, leading to cell death through multiple pathways. Cell death plays a crucial role in the development of cancer disease. Also, several ovarian diseases, including polycystic ovary syndrome and ovarian cysts, are associated with ER. OC cells have a well-developed ER system, but there are limited studies that analyze the mechanism of OC development and develop new therapeutic strategies from the ER, especially the Endoplasmic Reticulum Stress Response Pathway, we aim to explore the possible role of this response pathway in OC, summarize how the pathway leads to different patterns of cell death and to illustrate the challenges and possibilities of its targeted therapeutic applications, in an attempt to provide a new strategy of targeted drug delivery oriented towards the organelle-Endoplasmic Reticulum Stress Response Pathway-cell death for the treatment of OC. **Table 1** summarizes the abbreviation mentioned in the whole article.

**Table 1:** Acronym list.

Full English name	Abbreviation
Ovarian Cancer	OC
Endoplasmic Reticulum	ER
Endoplasmic Reticulum Stress	ERS
Unfolded Protein Response	UPR
Glucose-regulating protein 78	GRP78
Heat shock protein 70	HSP70
Inositol requiring enzyme 1	IRE1
Protein kinase RNA-like endoplasmic reticulum kinase	PERK
Activating transcription factor 6	ATF6
X-box binding protein 1	XBP1
Regulatory IRE1α-dependent decay	RIDD
Tumor necrosis factor receptor-associated factor 2	TRAF2
Apoptosis signal-regulating kinase 1	ASK1
c-Jun N-terminal kinase	JNK
B cell lymphoma-2	BCL-2
B-Cell Lymphoma-extra-large	BCL-xL
Immunoglobulin heavy-chain-binding protein	BiP
eukaryotic translation initiation factor 2α	eIF2α
C/EBP homologous protein	CHOP
Activating transcription factor 4	ATF4
basic-region leucine zipper	bZIP
Glucose-regulated protein 94	GRP94
Death-associated protein kinase 1	DAPK1
Transient receptor potential channel canonical 3	TRPC3
Reactive Oxygen Species	ROS
Nicotinamide Adenine Dinucleotide Phosphate	NADPH
Glutathione	GSH
Glutathione Disulfide	GSSG
NADPH oxidase 4	NOX4
Endoplasmic reticulum oxidoreductin-1	Ero1
Hexosamine Biosynthetic Pathway	HBP
Uridine diphosphate glucuronic acid	UDP-GlcUA
cellular Myc	c-Myc
neuroblastoma-derived myc	N-Myc
Mitochondrial DNA	mtDNA
BCL-2-associated X protein	Bax
Cytochrome c	cyt c
Damage-Associated Molecular Patterns	DAMPs
NLR family pyrin domain containing 3	NLRP3
Neutrophil-to-lymphocyte ratio	NLR
Platelet-to-lymphocyte ratio	PLR
C-reactive protein	CRP
Tumor Necrosis Factor	TNF
Interleukin-6	IL-6
Interleukin-8	IL-8
Interleukin-1beta	IL-1B
Interleukin-23	IL-23
Interleukin-24	IL-24
Protein Disulfide Isomerase	PDI
Coactivator-associated arginine methyltransferase 1	CARM1
Kelch-like ECH-associated protein 1	Keap1
ER-resident endoplasmic oxidoreductin-1 alpha	Ero1α
DNA Damage Inducible Transcript3	DDIT3
Inositol 1,4,5-triphosphate	IP3
Immunohistochemistry	IHC
Cysteinyl aspartate specific proteinase	Caspase
Bcl-2 interacting mediators of cell death	Bim
Melatonin receptor 1	MT1
Ectonucleoside Triphospate Diphosphohydrolase 5	ENTPD5
Nucleoside triphosphate diphosphohydrolases	NTPDase
Immunogenic Cell Death	ICD
Calreticulin	CRT
Transient receptor potential vanilloid 1	TRPV1
Cisplatin	DDP
3-methyladenine	3-MA
Toll-like receptor 4	TLR4

## 2 Endoplasmic reticulum stress and unfolded protein response

**Figure 1:**
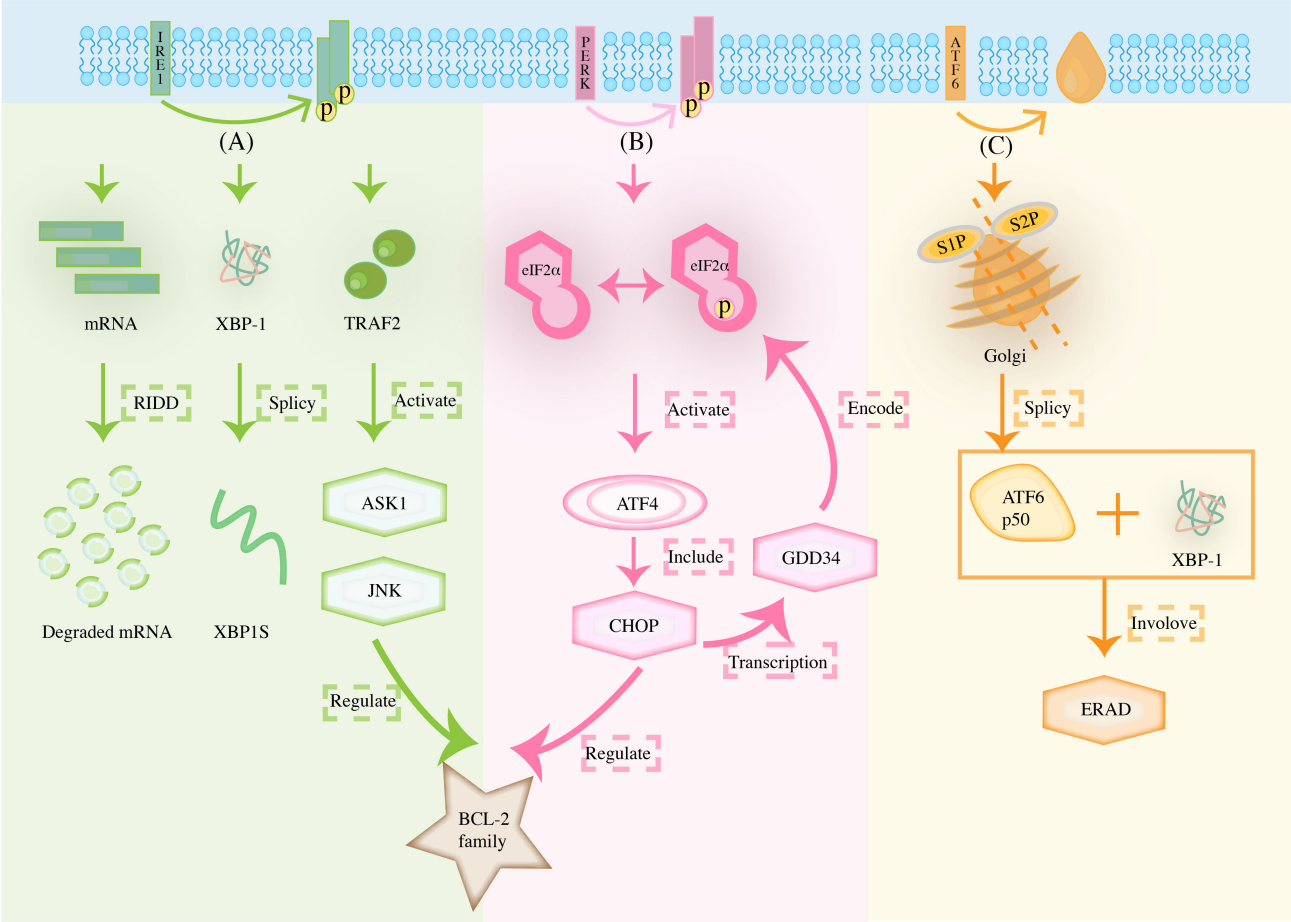
The unfolded protein response determines cell fate through **(A)** IRE1, **(B)** PERK, and **(C)** ATF6 pathways. (The stimulation of microenvironmental stress can impair protein folding in the ER, including changes in altered redox levels in the reticulum lumen, blocked glycosylation of protein junctions, and imbalance in Ca^2+^ regulation, amongst others. In response to the accumulation of ER misfolding proteins, the UPR is initiated by three transmembrane ER proteins: IRE1, PERK, and ATF6).

ER is a unique membranous structural organelle that can participate in biosynthesis, lipid metabolism, calcium homeostasis, preside over protein folding, and secrete intracellular proteins. It synergizes with other organelles, including mitochondria and lysosomes, to crosstalk various signals (8). The existence of a protein folding state is a physiological feature unique to the ER lumen. When there are changes in the microenvironment, such as altered redox levels in the reticulum lumen, blocked glycosylation of protein junctions, and imbalance in Ca^2+^ regulation, the folding of proteins in the ER lumen drives away from correct folding and thus towards misfolding (9). Moreover, the accumulation of misfolding induces ERS, which triggers a cellular stress response mechanism called UPR to reprogram gene transcription, mRNA translation, and protein modification, thereby alleviating the load on the unfolded or misfolded proteins. ER is involved in the maintenance of cellular homeostasis through this series of stress response pathways to restore the normal cellular microenvironment. At present, the most essential protein in this pathway is glucose-regulating protein 78 (GRP78). GRP78, a member of the heat shock protein 70 (HSP70) family, corrects protein folding and increases GRP78 expression when ERS occurs (10). Furthermore, GRP78 binds to the unfolded protein, causing the dissociation of three sensors of the UPR [inositol requiring enzyme 1 (IRE1), protein kinase RNA-like endoplasmic reticulum kinase (PERK), activating transcription factor 6 (ATF6)] which then become free and activated. It then activates the UPR through their respective signaling pathways to affect cancer growth at many levels, including cell survival and death, angiogenesis, inflammation, antigen presentation, invasion, and metastasis (11). **Figure 1** summarizes how the unfolded protein response determines cell fate through IRE1, PERK, and ATF6 pathways.

### 2.1 The IRE1 pathway

The first sensor, IRE1, is a type I transmembrane protein with serine/threonine protein kinase and ribonucleic acid endonuclease activities at the cytoplasmic end. When the UPR is activated, the protein phosphorylates itself to form a dimer. After that, X-box binding protein 1 (XBP1) mRNA is cleaved catalytically to induce the activation of XBP1s for subsequent gene transcription processes, regulate protein degradation, and promote the adaptive survival of cells (12). However, sustained and high levels of autophosphorylation lead to higher order IRE1α oligomerization. Under these conditions, the RNA enzyme of IRE1α relaxes its specificity and endocytically degrades many mRNAs at the ER membrane encoding secreted proteins, a process known as regulatory IRE1α-dependent decay (RIDD). Furthermore, IRE1α recruits tumor necrosis factor receptor-associated factor 2 (TRAF2) to activate apoptosis signal-regulating kinase 1 (ASK1) and c-Jun N-terminal kinase (JNK) to form the IRE1α/TRAF2/ASK1/JNK complex, which down-regulates the expression of the anti-apoptotic genes B cell lymphoma-2 (BCL-2) and B-Cell Lymphoma-extra-large (BCL-xL), and activates Caspase-12 to induce apoptosis, and participates in the activation of intracellular inflammatory responses (13–16).

### 2.2 The PERK pathway

PERK is another important receptor during ERS; its amino end is located in the ER, which can sense the increase in the number of folded proteins, and the carboxyl-terminus end is located in the cytoplasm, which carries a serine/threonine protein kinase domain. As another type I ER-resident transmembrane protein, PERK often senses ERS with its own luminal structural domain. Usually, when the ERS condition persists, PERK dissociates from immunoglobulin heavy-chain-binding protein (BiP) and changes dimerization and autophosphorylation (16). Activated PERK phosphorylates serine 51st of eukaryotic translation initiation factor 2α (eIF2α) to inhibit protein translation to reduce the overload of proteins into the ER and alleviate the pressure on the folding of ER proteins. PERK activation initially leads to a protective cell survival response; however, sustained activation of the ERS process induces transcription of C/EBP homologous protein (CHOP), a transcription factor actively controlled by activating transcription factor 4 (ATF4), which is directly responsible for causing the expression of pro-apoptotic BCL-2 family members. Note that this event is critical for controlling the transition from survival to apoptosis. Phosphorylated eIF2α activates ATF4, which in turn acts on pre-target apoptotic genes such as GADD34 and CHOP, which can move to the nucleus, up-regulate its apoptotic target genes, and promote programmed cell death under ERS (17).

### 2.3 The ATF6 pathway

The third type of sensor, ATF6, is a type II transmembrane protein, which is often distributed on ER membranes in the form of zymogen, including ERS sensing lumen domain and cytoplasmic domain encoding the basic-region leucine zipper (bZIP) transcription factor (18). When ERS occurs, it is transferred to the Golgi as a vesicle to participate in subsequent movements. When full-length ATF6 (ATF6 p90) is transferred to the Golgi, ATF6 is sequentially cleaved by S1P and S2P to remove the transmembrane domain and intracellular domain. It, thereby, releases a fragment containing the alkaline leucine zip transcription factor (ATF6 p50), which later, together with XBP1, increases the transcription of related targets, such as BiP, glucose-regulated protein 94 (GRP94), p58(IPK)/DNAJC3. This, in turn, enlarges the size of the ER, increasing its protein-folding capacity and participating in ERAD pathway transcription (16). When ERS is persistently present, soluble ATF6 formed after protein hydrolysis up-regulates the expression level of death-associated protein kinase 1 (DAPK1) to induce BCL-1 phosphorylation and effective dislocation of BCL-2 to trigger autophagy (19). Accordingly, the sustained activation of the ATF6 pathway also strengthens vascular neogenesis and improves the immunogenicity of cancer cells.

The stimulation of microenvironmental stress can impair protein folding in the ER, including changes in altered redox levels in the reticulum lumen, blocked glycosylation of protein junctions, and imbalance in Ca^2+^ regulation, amongst others. In response to the accumulation of ER misfolding proteins, the UPR is initiated by three transmembrane ER proteins: IRE1, PERK, and ATF6. Note that under low levels of ERS, IRE1 undergoes autophosphorylation to form dimers. It also cleaves unconventional introns from XBP1 mRNA, which encodes XBP1s transcription when ligated, up-regulating ER protein folding and quality control components to facilitate adaptation. However, if over-activated by persistent ERS, IRE1α oligomerizes, and many mRNAs on the ER membrane are degraded, resulting in RIDD, leading to cell death. At the same time, the recruitment of TRAF2 regulates the involvement of ASK1, JNK in the control of BCL-2 family. PERK signaling controls ATF4 expression through phosphorylation of eIF2α, induces transcription of CHOP, and participates with the former in the control of the BCL-2 family. Meanwhile, persistent over-activation of ERS would prompt ATF4 to, in turn, act on pro-apoptotic genes such as GADD34. ATF6 translocates to the Golgi and is cleaved by site 1 and site 2 proteases, releasing the ATF6 p50 transcription factor into the cytoplasm, which then migrates to the nucleus where, together with XBP1, it participates in the transcription of the ERAD pathway.

ERS mediates the UPR primarily through three unfolded protein response pathways, and the respective signaling pathways mediated by the three sensors that cause dissociation of the UPR (IRE1, PERK, ATF6) influence OC development at many levels. Lin W et al. (20) discovered that OC cells with coactivator-associated arginine methyltransferase 1 (CARM1) were selectively sensitive to inhibition of the IRE1α/XBP1s pathway. In addition, the inhibition of the IRE1α/XBP1s branch alone or in combination with immune checkpoint blockade provided a therapeutic strategy for several cancer types, including OC, with frequent CARM1 overexpression. XBP1 is up-regulated in OC cell lines, and knockdown of XBP1 significantly inhibits cell proliferation and modulates oxidative stress to enhance the sensitivity of OC cells to H_2_O_2_ by increasing intracellular ROS levels (21). IRE1α-XBP1s is also involved in the regulation of OC stemness, and its activation also reprograms cancer-associated dendritic cells and T cells (22, 23). Meanwhile, the key function of IRE1 signaling pathway conduction in regulating OC cells has been established. At the same time, IRE1α inhibitors were discovered to affect cell growth/apoptosis in ovarian malignant cells via the XBP1-CHOP-Bim pathway after ERS (24). PERK phosphorylates the nuclear factor E2-related factor 2 (Nrf2), dissociating it from the Kelch-like ECH-associated protein 1 (Keap1) complex and giving it the ability to function as a transcriptional inducer of the protective antioxidant response. PERK activation also up-regulates ER-resident endoplasmic oxidoreductin-1 alpha (Ero1α) to promote protein folding in the ER, resulting in increased oxidative protein folding capacity, favoring cancer growth (3). However, the PERK signaling pathway is most closely related to apoptosis, probably since subject ATF4 expression is essential for activating apoptosis by regulating CHOP. Related literature (25) also confirmed that PERK branch activation and ATF4-mediated transcriptional induction of CHOP played a central role in forming OC cell apoptosis. Nevertheless, the three primary sensors (PERK, IRE1, and ATF6) and their downstream cascades all play different roles in ERS-induced apoptosis; however, the PERK pathway plays a central role (26–28). It suggests that ERS-UPR plays an important role in ovarian cell injury and ovarian cancer cell progression.

## 3 The inducible conditions of endoplasmic reticulum stress response pathway in ovarian cancer

### 3.1 Ca^2+^ homeostasis disruption

Ca^2+^ is a crucial and influential factor involved in cellular activities such as gene expression, protein synthesis, cell proliferation, and differentiation and is closely related to the ER (29). When exogenous factors stimulate the cells, a large amount of Ca^2+^ is released from the ER, causing ER dysfunction. Moreover, calcium homeostasis and signaling processes are the main support points for maintaining the normal function of the ER (30). Additionally, Ca^2+^ is often required to activate dependent molecular chaperones to stabilize protein-folding intermediates involved in ERS (29). Therefore, Ca^2+^ is an essential molecule in the homeostasis of the ER.

Dysregulation of calcium homeostasis has been suggested to serve as a marker of cancer cell development and plays a decisive role in many cancers, including OC. The intracellular Ca^2+^ concentration is dependent on the release of Ca^2+^ from the ER and extracellular Ca^2+^ intake. Its movement in and out of the cell is strictly controlled by a large number of calcium pumps, channel proteins, exchange proteins, and calcium-binding proteins. Note that extracellular Ca^2+^ determines cell proliferation and differentiation. The increase of intracellular basal Ca^2+^ and transient Ca^2+^ are involved in cell cycle change and cell proliferation. In addition, the characteristics of cancers are related to changes in specific aspects of the Ca^2+^ signaling pathway during proliferation. For example, increasing transient receptor potential channel canonical 3 (TRPC3)-dependent Ca^2+^ inflow can lead to the proliferation of OC cells (31). At the same time, ERS can induce an increase in intracellular Ca^2+^ concentration and the enhancement of autophagy, which stimulates Ca^2+^ in the ER to enter the cytoplasm to promote activation of autophagic responses. Therefore, it is possible that there is a bidirectional regulation of the complex network of ERS and Ca^2+^ cellular level regulation that affects signaling in cancer cells.

### 3.2 Intracellular accumulation of reactive oxygen species

Reactive Oxygen Species (ROS) is a type of free radical. In general, free radicals, including ROS and RNS, play a regulatory role in Endoplasmic Reticulum Stress Response Pathway. Cell growth, proliferation, and apoptosis depend on the intracellular redox state in the cell, whose balance is precisely dominated by ROS. Moreover, it is widely involved in signaling oxygen radicals and their derivatives, regulating cellular responses to external stimuli to determine the fate of the cell. ROS are formed *in vivo* by the mitochondrial electron transport chain and Nicotinamide Adenine Dinucleotide Phosphate (NADPH) oxidase. Under normal conditions, ROS is at a low level in the body. If there is an excess of ROS, it will damage the function of the ER and induce the unfolded protein reaction and ERS (32). In addition, Liu ZW et al. (33) suggested this may be an important mechanism leading to apoptosis. Shi WZ et al. (34) and Wei J et al. (35) also came to a similar conclusion. The interaction between ROS and ER is mediated by several characteristic factors and signaling pathways, such as Glutathione (GSH), Glutathione Disulfide (GSSG), NADPH oxidase 4 (NOX4), and Ca^2+^, amongst others (36). At the same time, the increased production of ROS may directly promote the up-regulation of ERS-related proteins (ATF6, GRP78, XBP1s), which play an anti-cancer role as a medium (37).

### 3.3 Hypoxia

Hypoxia is a common feature of the cancer microenvironment, disrupting ER homeostasis and causing pressure in this compartment. Most proteins used in the extracellular space require disulfide bonds for folding and stabilization. Disulfide bonds introduced rapidly during protein synthesis can occur without oxygen. However, disulfide bonds introduced during post-translational folding or isomerization are highly oxygen-dependent. Therefore, oxygen-dependent disulfide bond formation after translation leads to hypoxia and induces ERS (38). May D et al. (39) obtained similar conclusions and further discussed that in physiological theory. In addition to not other proteins in the relay of disulfide formation, endoplasmic reticulum oxidoreductin-1 (Ero1)-L alpha, which is necessary for disulfide bond formation and protein folding, is also regulated by hypoxia and involved in cancer progression. Solid cancers exhibit a heterogeneous microenvironment, often characterized by concentration limitations of O_2_, glucose, and other nutrients, in which hypoxic cells deprived of serum lipids fail to extend their ER appropriately. This can lead to IRE1-dependent cell death, which can be reversed by the addition of unsaturated lipids, suggesting that the reduced content of unsaturated lipids in hypoxic cells triggers ERS by restricting the expansion of the ER (40).

### 3.4 Others

**Figure 2:**
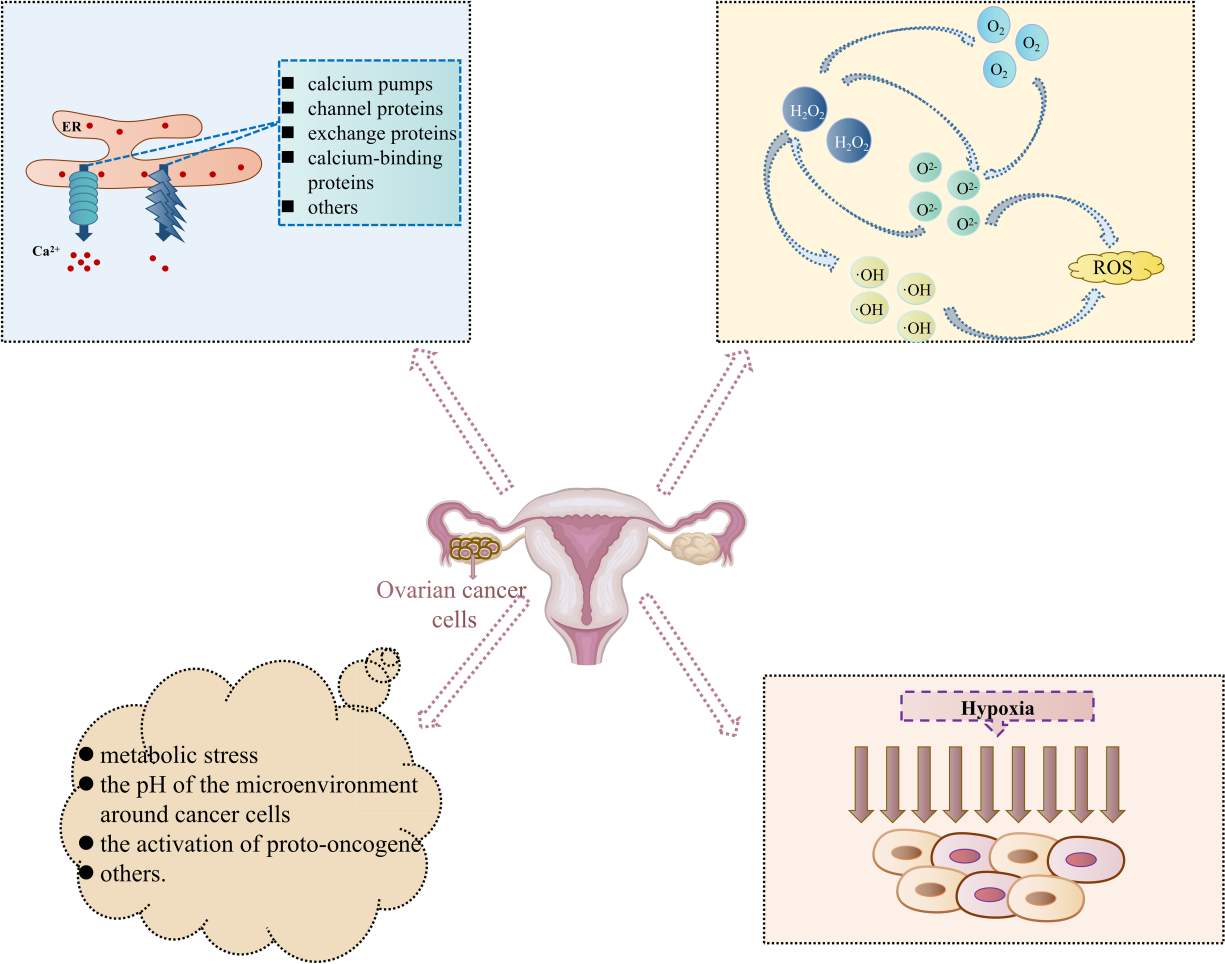
Inducible conditions related to ovarian cancer cell progression-induced Endoplasmic Reticulum Stress Response Pathway. (In the special cell environment of cancers, changes in internal and external environments such as Ca^2+^ homeostasis disruption, intracellular accumulation of reactive oxygen species, hypoxia, and others, provide a good environment for the occurrence and development of Endoplasmic Reticulum Stress Response Pathway and then participate in different cell biological processes such as abnormal proliferation, differentiation, and apoptosis of ovarian cancer cells).

Metabolic stress is characterized by insufficient or excessive nutrient supply compared to typical cellular energy requirements. It disrupts ER homeostasis since glucose and glutamine are mediated by the Hexosamine Biosynthetic Pathway (HBP) to control uridine diphosphate glucuronic acid (UDP-GlcUA). Furthermore, it is essential for N-linked glycosylation and protein folding in the ER (41, 42). Glucose restriction affects ATP production, blocking the energy sources and phosphate donors required for protein folding in the ER (43). In addition, the pH of the microenvironment around cancer cells may also be present to activate ERS. Aerobic glycolysis of central metabolic pathways can reduce lactate in the surrounding microenvironmental pH. In contrast, low pH may trigger the UPR of different signaling pathways by disrupting intracellular calcium homeostasis or inducing ROS overproduction (3). In oncologic diseases, different degrees of extrinsic factors (hypoxia and nutritional deficiencies, to name a few) cause ERS and trigger the UPR. However, simultaneously, there are still intrinsic factors of oncogene activation that induce ERS. It has been discovered that the activation of proto-oncogene MYC/cellular Myc (c-Myc) can activate ERS. c-Myc and neuroblastoma-derived myc (N-Myc) activated the PERK/eIF2α/ATF4 signaling pathway in the UPR, which led to an increase in cell survival by inducing cytoprotective autophagy. Meanwhile, the inhibition of PERK significantly reduced Myc-induced autophagy and cancer formation (44). **Figure 2** summarizes all the inducible conditions related to OC cell progression-induced Endoplasmic Reticulum Stress Response Pathway.

## 4 Ovarian cancer cell death mediated by endoplasmic reticulum stress response pathway

**Figure 3:**
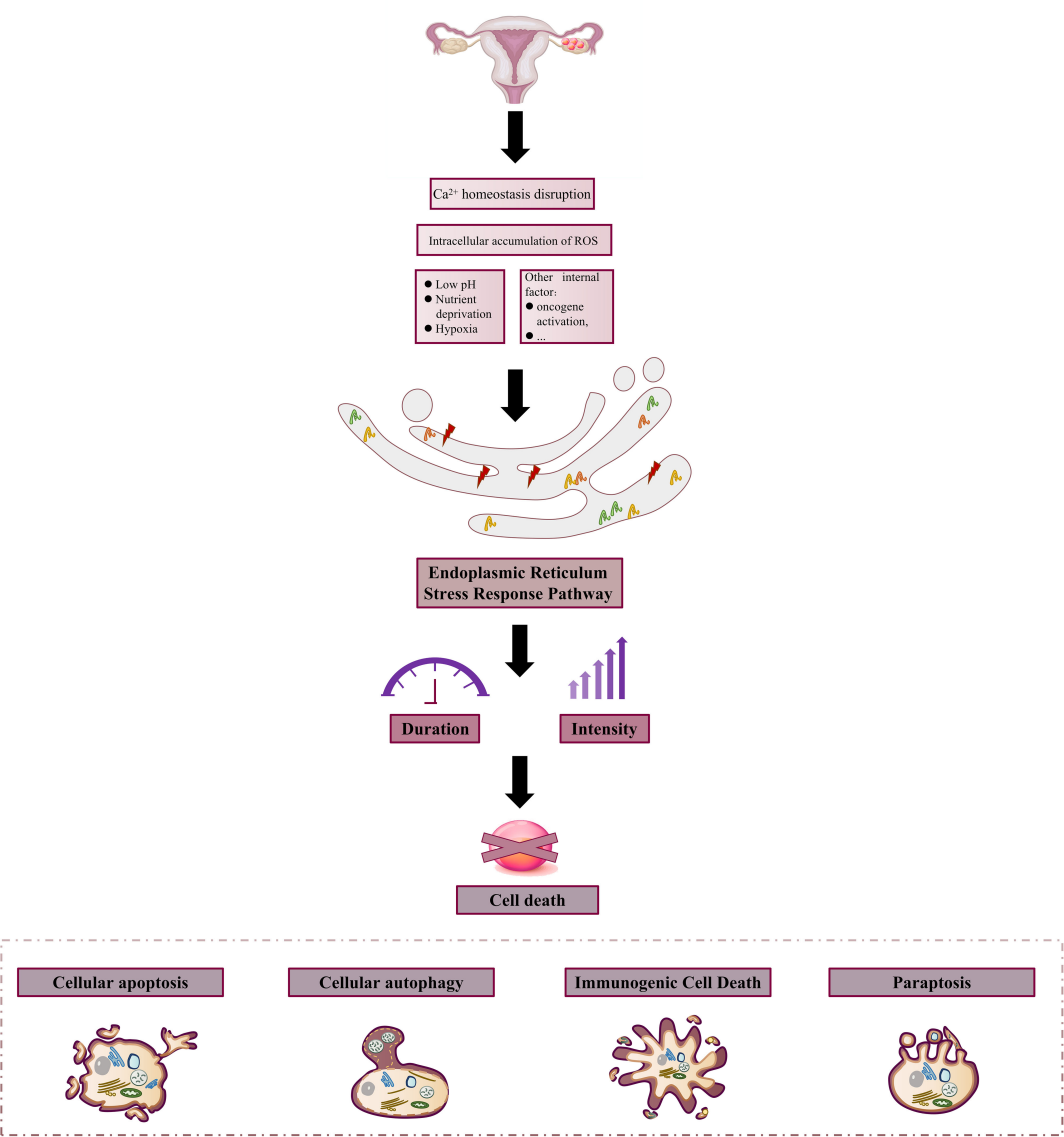
Constant stimulation induces ovarian cancer cell death. In the harsh external microenvironment characterized by hypoxia, nutritional deprivation, low pH, Ca^2+^ homeostasis disorder, and ROS over-production, or internal environmental changes such as the activation of oncogenes due to certain changes, the protein folding ability of the ER in cancer cells and infiltrating immune cells is changed. At the same time, the condition induces the onset of Endoplasmic Reticulum Stress Response Pathway and triggers cell death due to the long duration and intensity of stress.

In OC, changes in the internal and external environments, such as hypoxia and Ca^2+^ homeostasis disruption; oxidative stress and inflammation in ERS; and the activities of different molecular and signaling pathways provide a good basis for the Endoplasmic Reticulum Stress Response Pathway to act on OC cells, which can then be involved in different cellular biological processes to mediate the survival and death of the cells. Upon stimulation, cells first usually respond to this stressful state to restore their normal physiological functions and maintain proteostasis through negative feedback regulation; however, when the stimulus is too strong or lasts too long beyond the adaptive regulation of the ER, it can lead to a serious imbalance in ER homeostasis, resulting in cell death. It is widely accepted that exposure to sustained external stimuli is a factor marker specific to cancer cells, as multiple metabolic and oncogenic abnormalities in the cancer microenvironment disrupt protein-folding homeostasis in malignant cells and infiltrating immune cells. Therefore, a prolonged and intense Endoplasmic Reticulum Stress Response Pathway is often present in ovarian tissues to induce cancer cell death (**Figure 3**).

### 4.1 Cellular apoptosis

Three signaling pathways are inextricably linked to apoptosis: the Endoplasmic Reticulum Stress Response Pathway, the mitochondrial pathway, and the death receptor pathway, especially the first one. The Endoplasmic Reticulum Stress Response Pathway is mediated by changes induced by the dissociation of three sensors, IRE1, PERK, and ATF6, as promoters, causing cells to be unable to overcome the stress conditions, disrupting the intracellular homeostasis and inducing the CHOP, Caspase-12, and JNK signaling pathways to promote apoptosis.

#### 4.1.1 The CHOP signaling pathway

CHOP, called DNA Damage Inducible Transcript3 (DDIT3), is a transcription factor specific to ERS, and its increased expression is thought to be a marker of apoptosis initiated by ERS. CHOP is involved in the apoptosis of cancer cells, mainly through excessive or persistent stress. In addition, UPR down-regulates the pro-survival gene BCL-2 by enhancing the expression of CHOP genes and up-regulates the expression of pro-apoptotic genes Bad/Bak. It also binds to inositol 1,4,5-triphosphate (IP3) receptor through Ero1α, induces Ca^2+^ release, and activates calcium-dependent protein kinase to induce apoptosis. Li DY (45) examined the expression of CHOP in ovarian benign and malignant cancer tissues by Immunohistochemistry (IHC) from 40 patients with ovarian serous adenoma undergoing initial cancer cell reduction and 30 patients with ovarian serous cystadenoma. The results suggested that the positive rate of CHOP in ovarian serous adenocarcinoma was lower than that in ovarian serous cystadenoma, which may be related to the inhibition of the ERS-induced apoptosis pathway. Gao R et al. (46) discovered that the PERK-eIF2α-ATF4 pathway was the most crucial for the expression of CHOP proteins and suggested that the failure of the PERK-ATF4-CHOP pathway to be successfully activated to induce apoptosis may be one of the essential reasons for cisplatin resistance in OC cells. In addition, the PERK pathway mediates apoptosis mainly through the induction of CHOP by the downstream transcription factors of PERK (ATF4, ATF6, especially the first type). CHOP mRNA can modulate CHOP instantaneously, making it more likely to be phosphorylated by p38MAPK after transcription, so CHOP’s pro-apoptotic activity increases. Since the downstream target of the IRE1-TRAF2-ASK1 signal transduction pathway is p38MAPK, CHOP phosphorylation by p38MAPK may be the link between PERK and IRE1 signaling pathways. The genetic study has found that the loss of CHOP function can protect cells, and the gain of CHOP function can increase the sensitivity of cells to foreign stress, so that the function of the ER is more easily disturbed (47). These results demonstrate that drugs that modulate CHOP expression can be involved in the development and prognosis of apoptosis in OC cells and that CHOP is a potential target for the treatment of oncological diseases.

#### 4.1.2 The Caspase signaling pathway

The Caspase pathway is also a significant pathway for the induction of apoptosis (48). The pathway is mainly divided into endogenous and exogenous pathways, Caspase, among which the endogenous pathway is mainly related to the activation of Caspase-9. Meanwhile, the exogenous pathway is mainly related to the activation of Caspase-8. However, it has been reported in recent years that the activation of Caspase-12 is most closely related to the Endoplasmic Reticulum Stress Response Pathway. The activation of the three pathways eventually leads to the activation of Caspase-3 and cell apoptosis. Caspase-12 is the specific mediator of the ERS apoptotic pathway and the initiation protein of the ERS apoptotic pathway. As the only member of the Caspase family located on the outer membrane of the ER, Caspase-12 can only be activated in the Endoplasmic Reticulum Stress Response Pathway to initiate the apoptotic pathway, which occurs as a cascade of subsequent Caspase family reactions (49). Thus, by regulating apoptosis, proteasogen becomes a proteolytic enzyme with biological activity and hydrolyzes in the next step. Moreover, dividing the proteins necessary to maintain the normal structure and biological activity of cells destroys the normal structure of cells and intracellular DNA and eventually causes cell death (50). It has also been revealed in the literature (51) that activation of ERS to mediate apoptosis mainly depends on regulating Caspase-12. It was discovered that Paeonol (52) can increase the protein expression levels of CHOP, cleaved Caspase-12, and cleaved Caspase-3. It also promotes the release of Ca^2+^ from the ER into the cytoplasm and SKOV3 cells apoptosis. Rezghi Barez S et al. (53) conducted experiments on the induction of apoptosis of SKOV3 cancer cells by miR-30c-2-3p, and the results demonstrated that the short non-coding RNA regulating gene expression could block the decrease of XBP1 mRNA expression. It moderately up-regulates the expression of CHOP and BCL-2 interacting mediators of cell death (Bim) proteins. In subsequent simulation studies, it was concluded that apoptosis was induced by activation of Caspase-12, Caspase-3, and increased Bax/BCL-2 ratio. This also suggests the critical role of the Caspase-12 pathway in activating ERS-guided apoptosis in OC.

#### 4.1.3 The JNK signaling pathway

Along with the CHOP and Caspase-12 pathways, the JNK signaling pathway, which is activated during ERS, is thought to be another pathway that promotes apoptosis. Maira Smaniotto Cucielo et al. (54) discovered that the significant anti-cancer effect of melatonin on OC cells was dependent on affecting the expression level of the JNK pathway inhibiting the melatonin receptor 1 (MT1) receptor to enhance apoptosis. Notably, the regulation of the JNK signaling pathway to activate ERS to induce apoptosis is currently a hot topic. The JNK signaling pathway is closely related to IRE1α. It has been discovered in the literature (55) that ERS can phosphorylate JNK and activate c-Jun through IRE1α. At the same time, sustained activation of JNK-c-Jun signaling induces apoptosis induced by activation of the pro-apoptotic protein Bax and inactivation of the pro-survival factor BCL-2. Moreover, IRE1 is phosphorylated in the cytoplasm to stimulate the activation of TRAF2, which in turn phosphorylates and activates ASK1, ultimately JNK (56). In addition, inflammation, oxidative stress, and other stimuli in the cancer microenvironment can activate JNK expression, and activation of the JNK pathway can, in turn, regulate inflammation, oxidative stress, and responses in the cancer microenvironment. Pan X et al. (57) discovered that traditional Chinese medicine preparations could play an inhibitory therapeutic role in OC by regulating the activation of the JNK signaling pathway and improving the cancer inflammatory microenvironment. Meanwhile, activated JNK can promote the expression of Caspase-3 and other apoptosis-related genes and further activate the death receptor or mitochondrial pathway to induce apoptosis (58). It was also discovered that increased apoptosis in OC cells is apparently associated with sustained activation of the JNK/p38 MAPK signaling pathway (59). Several studies have explored drugs and compounds that regulate the JNK pathway, such as Protodioscin, Sertraline, and Sevoflurane, which can control apoptosis and exert anti-cancer effects (59–62).

**Figure 4:**
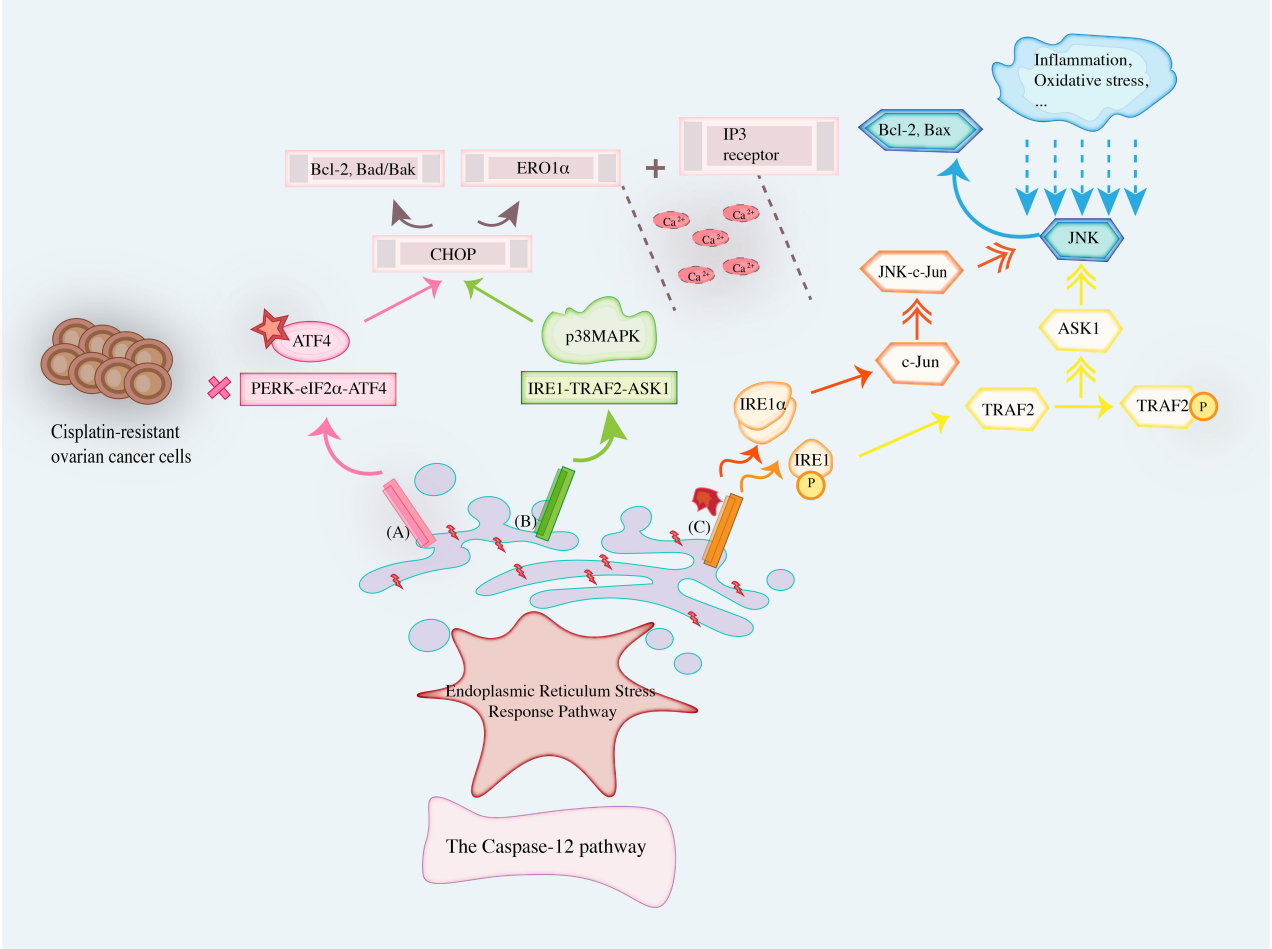
Cellular apoptosis in ovarian cancer induced by Endoplasmic Reticulum Stress Response Pathway.

Therefore, Endoplasmic Reticulum Stress Response Pathway is mediated by the changes caused by the dissociation of IRE1, PERK, and ATF6 sensors as promoters, which cause OC cells to be unable to overcome the stress conditions, destroy cell homeostasis, and apoptosis. UPR can regulate the expression of BCL-2 and Bad/Bak by enhancing CHOP gene expression, and induce Ca^2+^ release by binding ERO1α to IP3 receptor. At the same time, PERK-ATF4-CHOP and IRE1-TRAF2-ASK1 signaling pathways affect ATF4 and p38MAPK to regulate CHOP initiation apoptosis, respectively. Caspase-12 is the specific mediator of the ERS apoptotic pathway and also the initiation protein of the ERS apoptotic pathway. As the only member of the Caspase family located on the outer membrane of the ER, Caspase-12 can only be activated in the ERS pathway to initiate the apoptotic pathway, and subsequent cascades of Caspase family occur. The JNK signaling pathway is closely related to IRE1α. ERS can phosphorylate JNK and activate c-Jun through IRE1α, while sustained activation of JNK-C-Jun signaling can lead to activation of pro-apoptotic protein Bax and inactivation of pro-survival factor BCL-2 to induce apoptosis. At the same time, IRE1 also underwent phosphorylation changes in the cytoplasm to stimulate the activation of TRAF2, which in turn phosphorylated and activated ASK1 and finally JNK. **Figure 4** summarizes cellular apoptosis in OC induced by Endoplasmic Reticulum Stress Response Pathway.

### 4.2 Cellular autophagy

Cellular autophagy is an effective cellular response to the environment and a vital repair mechanism in which many cytoplasmic macromolecules, misfolded proteins, and damaged organelles are transported to autophagic lysosomes and hydrolyzed by the lysosomes to produce amino acids, nucleotides, sugars, and adenosine triphosphate for final recycling (63). ERS often uses autophagy as an intermediate mediator to fulfill the metabolic needs of cells (64). Initially, autophagy was considered a pure cancer suppressor mechanism since basic autophagy-related genes such as BECN1 (ATG6) have allelic deletions in the human breast, ovarian, and prostate cancers (63). However, recent studies suggested that autophagy plays a different role in different stages of cancer development and other environments. In the early stage of cancer development, autophagy degrades damaged organelles, alleviates oxidative stress, reduces DNA damage, prevents genomic instability, and inhibits cancer development. In the late stage of cancer development, microenvironmental stress affects the survival of cancer cells through autophagy (65, 66). Furthermore, UPR can relieve ERS, help misfolded and unfolded proteins rebuild their physiological structure, and activate autophagy. When ERS stimulation persists, the UPR is unable to remove the unfolded protein, eventually inducing autophagy to reduce ER swelling due to the increasing number of protein fragments (63). Therefore, there is an inextricable link between Endoplasmic Reticulum Stress Response Pathway, cellular autophagy, and cancer disease.

ERS inducers can activate autophagy, and autophagy can balance ER expansion induced by ERS and promote cell survival. Similarly, ERS significantly enhances the apoptosis of OC cells. At the same time, autophagy inhibits the apoptosis of OC cells; hence, it is vital to explore the relationship between the two in the targeted therapy of OC (67). Previous studies on cancer drug resistance have discovered that cancer drug resistance is mainly related to the following molecular mechanisms: high expression of Multidrug Resistance (MDR) genes, insufficient apoptosis, enhanced DNA damage repair, and signaling pathways such as drug transport. However, with the depth of the study, high expression of anti-apoptotic proteins such as BCL-2, which leads to the apoptotic escape of cancer cells, has been considered another significant mechanism (68). The occurrence of autophagy is often accompanied by the appearance of apoptosis. The BCL-2 protein family, which is mainly involved in the regulation of the mitochondrial apoptosis pathway and has been discovered to be closely related to the ER in recent years, may be a conductive factor between Endoplasmic Reticulum Stress Response Pathway, autophagy, and cancer disease. In addition, BCL-2 proteins localized to the ER may sense the signal of transmitter damage and transmit death signals to mitochondria or directly regulate apoptosis at the ER level (69). Bax and Bak act at the ER membrane to activate IRE1a signaling and provide a physical link between the core apoptotic pathway members and the UPR (70). Therefore, the BCL-2 protein family would regulate ER stabilization and mediate ERS. While autophagy is a cellular defense mechanism against misfolded proteins, and UPR regulates this regulation of autophagy, BCL-2 located in the ER can increase cytoplasmic Ca^2+^ by influencing calcium ion agonists, inducing the autophagy process (71). Hu HC et al. (72) discovered that the positive expression of BCL-2 in OC tissues was higher than in adjacent normal tissues. Wei W et al. (73) studied the expression and significance of BCL-2 protein in high-grade serous carcinoma of the ovary. The authors discovered that the difference in the positive expression rate of BCL-2 protein in the four groups of normal, benign, borderline, and malignant groups was statistically significant [P < a (0.05)]. Note that the influencing factors of the expression of BCL-2 protein were mainly platinum resistance, ascites cancer cells, and serum CA125 level (P < 0.05), in which platinum resistance was positively correlated with the intensity of BCL-2 protein expression. In contrast, ascites cancer cells and serum CA125 level were negatively correlated with the intensity of BCL-2 protein expression. Wang LN et al. (74) studied the effects of piceatannol on the proliferation, migration, and invasion of human OC SKOV-3 cells and discovered that after the intervention of piceatannol, the expression of BCL-2 protein in SKOV-3 cells was significantly decreased. Meanwhile, the expression of Bax and cleaved Caspase-3 protein was significantly increased. This also indicates that the BCL-2 protein family is closely related to OC, which enhances the reliability of ERS mediated by the BCL-2 protein family and autophagy on the core of OC cells. ER is the major intracellular Ca^2+^ reservoir; Ca^2+^ is the main molecule for the exchange of information between mitochondria and ER and is closely related to the activation of autophagy reaction. BCL-2 has a regulatory effect on intracellular Ca^2+^ homeostasis. This can mediate ER Ca^2+^ uptake and reduce the oxidative damage of the ER membrane (75). It is also mentioned above that Ca^2+^ is a related factor of ERS caused by OC injury, so it follows that inducing a relevant response of the BCL-2 protein family exacerbates the changes in Ca^2+^ levels. In addition, it further enhances ERS, which interacts with cellular autophagy in this way for modulation of the cancer cells.

### 4.3 Immunogenic cell death

Immunogenic cell death (ICD), a form of regulated cell death (RCD), activates adaptive immune responses in the presence of normal immune function, and specific cancer therapies performed in this way in recent years offer the potential to convert non-immunogenic inducers of cell death into potent ICD inducers. It offers improved cancer vaccination strategies and opens up prospects (76). Motolimod, a novel TLR8 agonist, promotes intracellular ICD in combination with adriamycin treatment for the integrated development of chemo-immunotherapy in OC (77). An immunogenic cell death-associated gene landscape using nine genes (ERBB2, RB1, CCR7, CD38, IFNB1, ANXA2, CXCL9, SLC9A1, and SLAMF7) to construct an ICD-associated prognostic signature predicts overall survival and immune infiltration status in OC (78). As a stress response of cancer cells, ICD not only activates anti-cancer immunity in the body, but also plays a unique role in different fields such as chemotherapy, radiotherapy, targeted therapy, and oncolytic virus (79). This suggests that ICD is closely related to OC. ERS is partly responsible for changes in the distribution of ER chaperon Calreticulin (CRT) cells, and the Endoplasmic Reticulum Stress Response Pathway regulates the release and binding of CRT to human ovarian cells and plays a role in ICD dependence (80). Bi F et al. (81) discovered that Benzenesulfonamide, a mitochondrial uncoupler, activates ERS sensors, resulting in ICD and anticancer immunity. This suggests a good feasibility of using Endoplasmic Reticulum Stress Response Pathway as a breakthrough to regulate ICD-induced cell death.

### 4.4 Paraptosis

Paraptosis, a non-apoptotic programmed cell death, often occurs in conjunction with apoptosis, although in contrast to apoptosis, apoptosis does not involve cysteinyl asparaginase activation or DNA breaks, and the main morphological features include swelling of the ER or mitochondria and cytoplasmic vacuolization (82). Accumulation of misfolded proteins in the lumen of the ER leads to the development of osmotic forces that result in water being withdrawn from the cytoplasm, leading to ER expansion; at the same time, ERS and expansion can result in release of ER Ca^2+^, i.e., in the overloading of mitochondria with mitochondria through the mechanism of intracellular Ca^2+^ fluxes located in the ER mitochondrial axis, which leads to mitochondrial expansion (83). Based on the rapid proliferation of cancer cells, inducing them to undergo cell death is an effective strategy for cancer therapy. Therefore, paraptosis, an atypical cell death mode, has a greater potential in cancer therapy. Since the morphological features of paraptosis are closely related to the ER, we conjectured that the Endoplasmic Reticulum Stress Response Pathway might be closely related to paraptosis. It has been shown that one of the inducing conditions for paraptosis to occur is a reduction in protein degradation (84). Inhibition of proteasome activity allows misfolded proteins to accumulate in the lumen of the ER, and the accumulation of proteins can lead to pressure overload of the cancer cell, ultimately causing cell death.

ERS occurs in OC cells exposed to hypoxia, disruption of Ca^2+^ homeostasis, and intracellular accumulation of ROS, which alter protein homeostasis and result in the accumulation of unfolded or misfolded proteins in the lumen of the ER. Subsequently, the 3 core UPR signaling pathways, IRE1, ATF6, and PERK, are induced. Endoplasmic Reticulum Stress Response Pathway orchestration is a highly dynamic process that may result in pro-survival and pro-apoptotic outputs, leading to adaptive restoration of homeostasis or cell death. Indeed, the decision of cell fate seems to depend on the duration and intensity of the stress of this process. The ER is enriched with a number of molecular chaperones that ensure the development of newly synthesized proteins, of which the ER chaperone GRP78 is relatively important. Under non-ER conditions, GRP78 binds to the inner end of the ER of these three molecules to maintain the inactivated state of the signal transduction factors when the ER is in a steady state; in the presence of the ERS and the UPR, GRP78 dissociates from the three transmembrane proteins and acts as a chaperone for the unfolded proteins to reduce the load caused by the unfolded or misfolded proteins (85). GRP78, which is now widely expressed in human cancers, was also found to correlate with poor patient prognosis with elevated GRP78 levels in ovarian tissue. It has been demonstrated that elevated levels of GRP78 in ovarian tissues correlate with poor patient prognosis. Moreover, OC section staining studies have demonstrated that GRP78 exhibits weak expression in cisplatin-sensitive ovarian cells and mediates cisplatin-induced senescence (20, 86). In addition, the former study also confirmed that another molecular chaperone, Protein Disulfide Isomerase (PDI), is also very abundant in ovarian tissue and is a potential biomarker of this disease, which can be used as a dual-prognostic marker (86). Since Endoplasmic Reticulum Stress Response Pathway core chaperones including GRP78 and PDI are actively expressed in ovarian tissues, using this as a starting point, we suggest that the main direction of ERS in OC cells is to induce the continuous activation of the three response pathways of the UPR with sustained environmental stimuli, which ultimately affects the life activities of ovarian cancer cells and leads to cell death. Abdullah TM et al. (80) came to similar conclusions and found that Endoplasmic Reticulum Stress Response Pathway is partly responsible for changes in the distribution of Calreticulin (CRT) cells, regulating to some extent the release and binding of CRT to OC cells and inducing cell death.

## 5 Treatment

**Figure 5:**
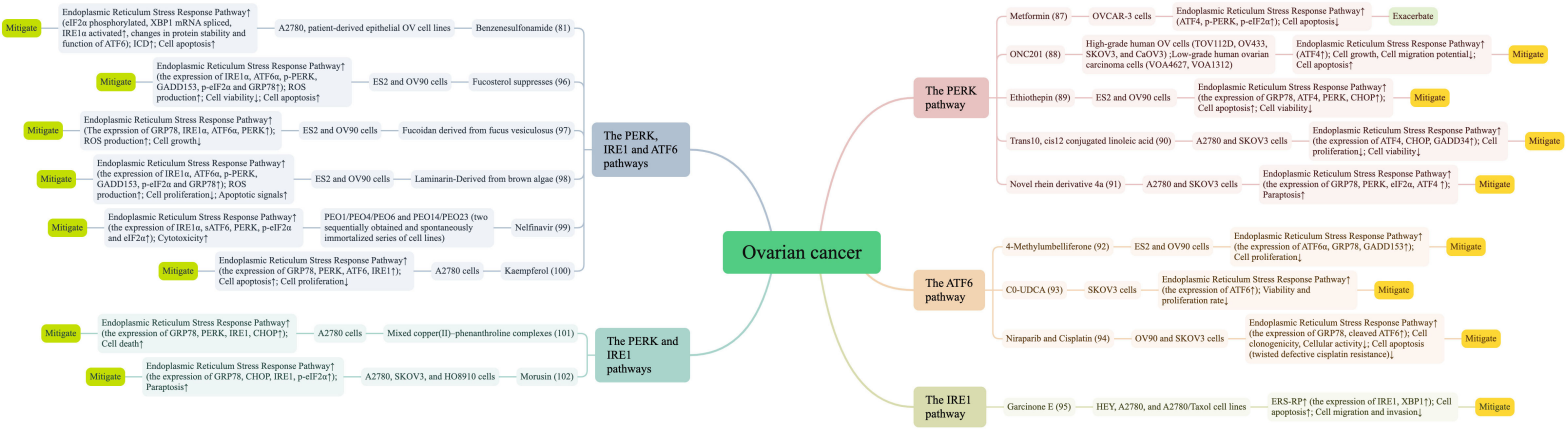
Literature summary of Endoplasmic Reticulum Stress Response Pathway and ovarian cancer treatment.

It is widely accepted that ERS and UPR play key regulatory roles in ovarian carcinogenesis and progression and may be important therapeutic targets for ovarian tissue and cancer-like diseases. Indeed, ERS remission and activation may play a role in the prevention, diagnosis, and treatment of OC. The Endoplasmic Reticulum Stress Response Pathway is used as a starting point to exert its anticancer effects by targeting cell death processes. In this regard, we further reviewed the current status of more comprehensive therapeutics targeting ERS signaling by summarizing the existing research literature in **Figure 5** (81, 87–102).

The main OC treatments include surgery, radiotherapy, chemotherapy and immune-targeted therapy. In this article, we found that other drugs can also affect OC through the Endoplasmic Reticulum Stress Pathway Response, such as metformin, antagonists, mifepristone, etc. Metformin can induce Endoplasmic Reticulum Stress Response Pathway with the starting point of inducing energy deprivation, especially with the PERK/eIF2α pathway. The study has shown that autophagy and PERK activation protect OC cells from metformin-induced apoptosis, and that inhibition of autophagy and PERK enhances the selective anticancer effects of metformin on OC cells (87). Another study (103) demonstrated that the concentration of glucose affects the action of metformin, characterized by low glucose enhancing the cytotoxicity of metformin on cancer cells and inducing apoptosis of OC cells through ERS triggered by the ROS/ASK1/JNK pathway. Epoxycytochalasin H, a fungal metabolite from Phomopsis, increased ROS levels in OC cell line A2780 cells, which not only triggered mitochondrial autophagy through mitochondrial damage and promoted apoptosis, but also elevated GRP78 expression suggesting mediation of ERS-related apoptosis (104). The novel transient receptor potential vanilloid 1 (TRPV1) antagonist DWP05195 induced apoptosis, and this type of apoptosis was most closely related to the Endoplasmic Reticulum Stress Pathway, with the core pathway of action being the ROS-p38-CHOP pathway (105). Saquinavir, an FDA-approved drug for the treatment of HIV, has also demonstrated promising results in the treatment of OC, inducing cell death in chemotherapy-sensitive and chemotherapy-resistant OC cells in a dual time- and dose-dependent manner, with the primary mechanism being that Saquinavir induces caspase-independent cell death by inducing ERS and autophagy (106). Studies on Saquinavir in recent years have shown that ERS may regulate cellular autophagy through the mTOR and Beclin 1 pathways, leading to a decrease in DDP sensitivity in SKOV3 cells, suggesting that ERS of the autophagic process in cancer cells is a potential target for enhancing the therapeutic efficacy of chemotherapy and reducing cancer drug resistance (107). Mifepristone has the ability to block the growth of OC cells, and in combination with proteasome or lysosomal inhibitors promotes mRNA translation, triggers the UPR, increases autophagic flux, and kills OC cells (108). Additionally, Lau TS et al. (109) reported that paclitaxel-induced ICD-related damage-related molecular patterns triggered significant anti-cancer response of paclitaxel-induced Toll-like receptor 4 (TLR4) signaling *in vivo* cancer vaccination assay. This emphasizes the vital role of ICD ability in the efficacy of chemotherapy drugs.

Also, the use of metal complexes in cancer therapy has attracted much attention, mainly because metals exhibit unique properties such as redox activity, metal-ligand interactions, structure, and bonding. The serendipitous discovery of the metal-based compound cisplatin by Barnett Rosenberg in 1965 was an outstanding breakthrough in the history of metal-based anticancer complexes and ushered in a new field of anticancer drug discovery (110). However, cisplatin therapy has certain limitations and new non-platinum-based drugs are entering the medical research horizon. The current research field of metal-based cancer drug development mostly introduces catalytic cancer drugs with organometallic Ru^II^, Os^II^, Rh^III^, and Ir^III^ complexes, with a regulatory mechanism that interferes with biochemical pathways (111). At the same time, metal-based cancer drugs have shown encouraging results in linking the Endoplasmic Reticulum Stress Response Pathway. For example, BOLD-100 (ruthenium complex sodium trans-[tetrachlorido-bis(1Hindazole) ruthenate(III)] (BOLD-100/KP1339), the most studied non-platinum metalloid anticancer drug, is also an inhibitor of stress-induced GRP78 up-regulation inhibitor, disrupts ER homeostasis and induces ER stress response (112). In OC, Copper (II)-phenanthroline complexes induce rapid cell death in OC cells through a UPR-linked mechanism (101). C0-UDCA (93), prepared from Cu(phen)2(OH2)](ClO4)2 (C0) and UDCA, showed higher efficacy than the precursors C0 and UDCA, and this new complex displayed anti-tumor effects on ovarian (SKOV-3) cancer cells at the ER level through activation of the UPR. In particular, the chaperone BiP, the pro-apoptotic protein CHOP, and the transcription factor ATF6 were upregulated in the presence of C0-UDCA.

Traditional medicine also plays a central role in the pathophysiological progression of disease, and herbal medicine, in particular, has been widely used alone or as a complementary approach to cancer treatment (113). We have found that TCM treatments are mostly based on the patient as a whole, with an eye to identifying and treating the symptoms, boosting the body’s positive qi, and enhancing the organism’s ability to resist external evils (114). This traditional therapy will modulate the Endoplasmic Reticulum Stress Response with diverse treatments such as herbal monomers, herbal combinations, and acupuncture. Artesunate, a semi-succinic derivative of artemisinin isolated from Artemisiae Annuae Herba, an essential drug for the treatment of malaria, inhibits PERK-eIF2α-ATF4-CHOP and IRE1α-XBP1 signaling pathways where activated to inhibit ERS. Banxia Xiexin Decoction reduces apoptosis by inhibiting activated transduction of the PERK-elF2α-CHOP apoptotic signaling pathway in ERS (115). Electroacupuncture of Fenglong point improved ERS stress and inflammatory response by inhibiting the expression of p-IRE1α and p-PERK and down-regulating the gene and protein expression of SREBP-1c (116). This affirms that traditional medicine mediates cell development survival by targeting ERS and UPR. It’s worth noting that herbal monomers occupy a central place in studies that specifically target OC. Kaempferol promotes apoptosis, reduces viability and proliferation of A2780 OC cells, and activates Endoplasmic Reticulum Stress Response Pathway-associated cytotoxic autophagy to achieve inhibition of OC progression using changes in Ca^2+^ levels as a starting point. The results of testing its effect on the sensitivity of OC cells to cisplatin (DPP) at the same time showed a strong inhibitory effect of kaempferol alone or in combination with DPP (100). Cucurbitacin-I affects both IRE1 and PERK signaling pathways leading to cell death and relies on the excessive ERS and CHOP-Bax and caspase-12-dependent ERS-associated apoptosis to induce cancer cell death forming a new anticancer strategy (28). Functional compounds α, β-thujone extracted from medicinal plants are conditionally stimulated by mitochondrial loss of function and metabolic alterations undergo Endoplasmic Reticulum Stress Response Pathway and cell death occurs via caspase-dependent intrinsic apoptosis pathway (117). Not only does quercetin induce ERS to target the p-STAT3/Bcl-2 axis involved in the mitochondrial apoptotic pathway, but also the experiment has shown that inhibition of ERS may not reverse quercetin-induced cell death; and that quercetin-induced ERS can concomitantly activate protective autophagy through activation of the p-STAT3/Bcl-2 axis (118). In addition, traditional medicine-related analogues and derivatives also play an important role. B19, a novel monocarbonyl analogue of curcumin, displays potent therapeutic power in epithelial OC. The cellular experiment (119) showed that B19 treatment induced autophagy in HO8910 cells, and the addition of 3-methyladenine (3-MA) inhibited autophagy, increased intracellular levels of misfolded proteins, and enhanced OC apoptosis. Pang HF et al. (91) discovered that novel rhein derivative 4a could stimulate the UPR of OC cells and induce paraptosis by up-regulating the expression of Bip78 and activating the PERK-eIF2α-ATF4 pathway. These findings could provide a new foundation for developing new strategies for treating OC.

The Endoplasmic Reticulum Stress Response Pathway is essential for cancer cells to adapt to rapid growth, hypoxia, nutrient deprivation, and chemotherapy. This response pathway has been revealed to play a two-fold role in cancer cell fate, in which the mechanism by which UPR signals switch from adaptive cellular protection to the threshold of apoptotic cell death or vice versa remains to be elucidated. Furthermore, metformin-induced apoptosis in OC cells is abrogated by autophagy and PERK activation; however, promoting apoptosis in OC cells via ERS induction has also been reported (90, 103). Quercetin induces protective autophagy in OC through ERS. However, it also aggravated DNA damage and led to typical apoptotic cell death; it acted as a promising radiosensitizer through p53-dependent ERS signaling (118, 120). These conflicting studies suggested that there may be a balance between cell death and survival depending on the degree and duration of drug stimulation. However, we can find from **Figure 5** that Endoplasmic Reticulum Stress Response dominantly tends to cell death more strongly in OC cellular processes, and most compounds are more adept at direct anticancer effects through Endoplasmic Reticulum Stress Response-induced cell death.

## 6 Discussion

Coordination of the Endoplasmic Reticulum Stress Response Pathway is a highly dynamic process that may result in pro-survival and pro-apoptotic outputs. Indeed, cell fate determination, in particular, whether cell death occurs, appears to depend on the duration and intensity of this process stress. The ER is enriched for several molecular chaperones that ensure proper folding of newly synthesized proteins. Endoplasmic Reticulum Stress Response Pathway has become a hot research field in cell biology.

ERS often occurs in cancer cells exposed to intrinsic factors and external triggers that alter protein homeostasis, accumulating unfolded or misfolded proteins in the ER lumen. It subsequently induces the three major UPR signaling pathways coordinated by IRE1, ATF6, and PERK, leading to adaptive restoration of homeostasis or cell death *in vivo*, known as Endoplasmic Reticulum Stress Response Pathway. The Endoplasmic Reticulum Stress Response Pathway is one mechanism that mediates various biological behaviors, such as cancer cell proliferation, autophagy, glycolytic energy supply, metastasis, and invasion. Note that it may play distinct roles at different stages of cancer-like diseases. The initial purpose of ERS is to maintain ER homeostasis. However, the role of long-term or severe ERS may be variable. Furthermore, the critical role of ERS coordination adaptation programs in regulating the fate of cancer cells has recently been highlighted in the field of OC research. After detecting ERS, the UPR coordinates adaptive programs to promote cancer cell survival. Nonetheless, the research has also made it clear that in the case of unmitigated ERS, effector mechanisms emanating from ERS can trigger and propagate danger signals in order to communicate a cell’s state of stress to its environment and/or induce cell death (121–123).

In growing cancers, changes in the pericellular microenvironment disrupt the activity of cancer cells inhabiting this environment. Harsh conditions have the ability to alter the protein folding capacity of the cancer cell ER, helping to up-regulate the incidence of ERS by the ongoing accumulation of misfolded or unfolded proteins within this organelle. The daily collaboration of the ER ensures rapid and precise control of Ca^2+^ signaling in space and time, and whether Ca^2+^ homeostasis is balanced or not is a key mediator of inter-organelle, and even inter-cellular, communication. With advances in the understanding of the role of Ca^2+^ homeostasis in cancer, we have found that there is a bi-directional modulation of the ERS and the complex network of cellular levels of regulation of Ca^2+^ in OC cells. Dysregulation of Ca^2+^ homeostasis disrupts dependent stable protein folding; ERS induces increased intracellular Ca^2+^ concentration and enhanced autophagy. ROS are central members in regulating various redox processes in the cell with the ability to cascade ERS with redox, and excessive ROS, enhance oxidative stress, impair ER function, induce Endoplasmic Reticulum Stress Response Pathway, cause DNA damage, and apoptosis in OC cells. Disulfide bonds introduced during post-translational folding or isomerization of proteins in the extracellular space are highly dependent on oxygen, while reduced unsaturated lipid content in hypoxic cells limits ER expansion. Changes in other factors encompassing metabolic stress, pH, and proto-oncogene activation also all manipulate Endoplasmic Reticulum Stress Response Pathway. Furthermore, in addition to changes in basic environmental factors, bi-directionally regulated inflammatory responses, differentially expressed GRP78, and PDI, a potential biomarker, all present key roles in Endoplasmic Reticulum Stress Response Pathway development in OC cells. It is precisely because of the continuous stimulation of these intracellular environment and factors that Endoplasmic Reticulum Stress Response pathway leads to more intense cell death in OC cell process.

Exploring new targets for the treatment of cancer diseases from organelles is a hot spot in the frontier clinical field. However, there are not many studies that deeply analyze the mechanism of OC occurrence and development and develop new therapeutic strategies from the ER itself, especially its functional characteristics. Therefore, it is crucial to deepen the application of Endoplasmic Reticulum Stress Response Pathway theory in OC from the perspective of cell biology at the organelle level. Human cells perform biological functions based on the synthesis of large amounts of proteins at any given time, and a certain amount of abnormal products, i.e., unfolded and misfolded proteins, can occur during this intense production process. The accumulation of unfolded proteins and misfolded proteins under different stimuli triggers the dynamic adjustment of the ER’s folding and degradation capacity, resulting in a series of subsequent reactions, which can be called the Endoplasmic Reticulum Stress Response Pathway. The Endoplasmic Reticulum Stress Response Pathway is involved in multiple stages of the cancer process by dominating specific cellular activities. Among them, the occurrence and development of OC are closely associated with different cellular processes dominated by Endoplasmic Reticulum Stress Response Pathway. Endoplasmic Reticulum Stress Response Pathway induces different modes of cell death, including cellular apoptosis, cellular autophagy, ICD, paraptosis and plays an important role in the treatment of ovarian tissue and OC disease. Therefore, by exploring the mechanism of Endoplasmic Reticulum Stress Response Pathway in regulating OC cell death, this review attempts to provide a new strategy of organelle-oriented precision targeting and drug delivery for OC treatment.

The study was limited to Endoplasmic Reticulum Stress Response Pathway and OC relevance. It is suggested that Endoplasmic Reticulum Stress Response Pathway is involved in the progression and treatment of OC, but more attention is needed to understand the key role of Endoplasmic Reticulum Stress Response Pathway in this disease. The direction of cell death process is mainly apoptosis and autophagy, and other involved types need to be further expanded. The amount of relevant literature is not sufficiently large, and the research on Endoplasmic Reticulum Stress Response Pathway tends to be a single-cell laboratory study, with a lack of actual multi-sample clinical studies. The safety of action, the reliability of targeted therapy, and the convenience of application still need to be further evaluated. The therapeutic direction is limited to the pure killing of cancer cells and lacks consideration of drug sensitivity in OC, versus thinking about the integrated treatment of cancer resistance.

Therefore, the future research direction can include the following contents. Capture the key factors of Endoplasmic Reticulum Stress Response Pathway and dig deep into the core role in OC from known research directions. Expand the scope of cellular processes to include cell division and cell proliferation in relation to entotic cell death, iron death, netotic cell death, and other modes of cell death. Laboratory linkage and attempts to carry out practical clinical observations and analyses of salient and meaningful therapeutic strategies. Promote meaningful research on Endoplasmic Reticulum Stress Response Pathway intervention in the process of platinum resistance in OC. Expand the application of Endoplasmic Reticulum Stress Response Pathway in oncological diseases and exploring the prospective application of Endoplasmic Reticulum Stress Response Pathway in other gynecological malignant diseases, such as endometrial cancer and cervical cancer, by drawing analogies.

## 7 Conclusion

In the present study, our main contribution is to combine the OC cell environment with various conditions that induce Endoplasmic Reticulum Stress Response Pathway, including Ca^2+^ homeostasis disruption, intracellular accumulation of ROS, hypoxia, and others. These factors not only directly stimulate Endoplasmic Reticulum Stress Response Pathway changes, but also may simultaneously combine oxidative stress, inflammatory response and other changes to bi-directionally regulate the extent to which the Endoplasmic Reticulum Stress Response Pathway proceeds, thereby determining cell fate. From the perspective of different organelles such as cellular apoptosis, cellular autophagy, ICD, paraptosis and other mechanisms of cell death regulation, we reveal the independent organelle-ER in order to control the application of Endoplasmic Reticulum Stress Response Pathway in the study of OC, and discuss the current new strategy of Endoplasmic Reticulum Stress Response Pathway-targeted drug delivery based on cell death.

Although this can be used to summarize potential therapeutic approaches for targeting Endoplasmic Reticulum Stress Response Pathway to reduce or increase cell survival for OC, it is unclear how cancer cells trade-off between adaptive response survival and cancer-killing under excessive stress. Since it has become evident that the strategy of modulating cancer stress-induced cellular processes may achieve the best therapeutic effect, the current research direction theme is limited to laboratory cells and lacks animal experiments and subsequent clinical trials. Therefore, more careful research is required in the future in order to apply potential clinically targeted therapeutic agents as early as possible, with a view to paving the way for new methods of treating cancer-like diseases.
